# Frailty Assessment and Its Impact in Hospital Outcomes After Cardiac Surgery - A Prospective Observational Study

**DOI:** 10.21470/1678-9741-2023-0178

**Published:** 2026-09-10

**Authors:** Verônica Borges Muniz da Silva, Julia Granado Carraro, Camila Bottura, Daniella Alves Vento, Alfredo José Rodrigues

**Affiliations:** 1 Department of Surgery and Anatomy, Faculdade de Medicina de Ribeirão Preto, Universidade de São Paulo, Ribeirão Preto, São Paulo, Brazil; 2 Division of Cardiothoracic Surgery, Department of Surgery and Anatomy, Faculdade de Medicina de Ribeirão Preto, Universidade de São Paulo, Ribeirão Preto, São Paulo, Brazil

**Keywords:** Thoracic Surgery, Frailty, Heart Valve Diseases, Coronary Artery Bypass, Outcome.

## Abstract

**Introduction:**

Frailty increases the risk of adverse outcomes after cardiac surgery. However, there is no consensus on how to best assess it.

**Objective:**

To verify the prevalence of frailty in patients undergoing elective valve surgery or coronary artery bypass grafting (CABG) and if a more comprehensive assessment of the physical, psychological, and nutritional domains would better discriminate the frailty phenotype and its association with in-hospital mortality.

**Methods:**

Besides the criteria proposed by Fried, the Mini Nutritional Assessment, gait speed, Duke Activity Status Index, Dutch Exertion Fatigue Scale, and screening for depressive symptoms (Patient Health Questionnaire-9) were used.

**Results:**

Overall, considering Fried’s criteria, 43.6% of the patients were frail, and 49.6% were pre-frail. Of those considered frail, 51% were under 60 years old. Fried’s frailty phenotype showed no association with postoperative in-hospital mortality. Using additional variables to characterize frailty domains, it was possible to distinguish two groups (clusters) of patients. In cluster 1, 84.8% were frail according to Fried’s criteria (vs. 27.4% in cluster 2, P < 0.001), the rate of postoperative infection was higher (24.2% vs. 7.1%, P = 0.022), and in-hospital mortality was significantly higher (27.3% vs. 9.5%, P = 0.020).

**Conclusion:**

We observed a considerable proportion of patients with Fried frailty phenotype in patients undergoing CABG or valve surgery, many of them under 60 years old, but those frailty phenotypes were not associated with postoperative hospital outcomes. However, the association of frailty phenotype with higher rates of postoperative in-hospital mortality was evident when additional criteria were used to assess the domains associated with frailty.

## INTRODUCTION

**Table:** 

Abbreviations, Acronyms & Symbols	
CABG	= Coronary artery bypass grafting
DASI	= Duke Activity Status Index
DEFS	= Dutch Exertion Fatigue Scale
IPAQ	= International Physical Activity Questionnaire
MNA®	= Mini Nutritional Assessment
NYHA	= New York Heart Association
PHQ-9	= Patient Health Questionnaire-9

Frailty can be defined as a decrease in energy reserves and resistance resulting in increased vulnerability to adverse health-related events when the individual is exposed to endogenous or exogenous stressors. This condition is a consequence of the decline in function across multiple physiological systems in which the neuro-immuno-endocrine system, nutritional status, low-grade inflammation, and oxidative stress play a significant role^[^1^]^.

Even though the concept of frailty has been defined as an aging-related syndrome, frailty may be a result of underlying processes not necessarily linked to aging^[^2^]^. Therefore, frailty reflects biological and phenotypic age, and not only chronological age. Consequently, it may occur in non-elderly people, notably in those suffering from chronic diseases^[^3^]^. Additionally, unfavorable socioeconomic conditions may impose an additional burden^[^4^]^.

Frailty has been recognized as a risk for adverse postoperative outcomes in cardiac surgery^[^5^]^. However, there is no consensus on how to best assess it^[^6^]^, given that the effectiveness of the instruments to measure frailty for preoperative risk assessment before cardiovascular operations lack validation, especially in non-elderly patients.

Therefore, the present study was designed to verify the prevalence of frailty in patients undergoing elective valve surgery or coronary artery bypass grafting (CABG) and if a more comprehensive assessment of the physical, psychological, and nutritional domains would better discriminate the frailty phenotype and its association with in-hospital postoperative mortality.

## METHODS

The study was approved by the Research Ethics Committee of our institution (Process 10358/2019, register 3.190.303), and all participants of the study gave free and informed consent.

Between January 2019 and December 2020, candidates for elective isolated valve surgery or isolated CABG, regardless of their gender and older than 18 years, were recruited and prospectively followed during the postoperative hospital stay. Patients with disability resulting from orthopedic conditions or stroke or those with cognitive impairment were excluded from the study. All operations were performed through a sternotomy using a cardiopulmonary bypass.

The presence of the frailty phenotype was evaluated according to Fried et al.^[^7^]^: a) unintentional weight loss was defined as weight loss > 4.5 kg or > 5% of body weight, in the last year; b) self-reported fatigue (“exhaustion”) was assessed according to the answer to the question: “Do you feel that you have to make an effort to do usual tasks?”; c) handgrip strength was evaluated using a manual dynamometer (Jamar®); d) the level of physical activity was assessed using the International Physical Activity Questionnaire (or IPAQ), short version, and scores consistent with “irregularly active B” or “sedentary” were used to score frailty; e) depressive symptoms were assessed using the Patient Health Questionnaire-9 (PHQ-9); f) the gait speed (“slowness”) test was performed according to standardized instructions: the patient was instructed to walk at a comfortable pace until few steps past a five-meter mark, beginning each trial on the word “go”; the timer was started with the first footfall after the zero-meter line and stopped with the first footfall after the five-meter line. Three trials were repeated, allowing sufficient time for recuperation between trials; “low gait speed” was considered if the five meters were walked in more than six seconds.

In addition to Fried’s criteria, we also assessed fatigue using the Dutch Exercise Fatigue Scale^[^8^]^, and the physical activity level using the Duke Activity Status Index (DASI). Preoperative hemoglobin and the Mini Nutritional Assessment (MNA®) were used to assess the nutritional state.

### Statistical Analysis

Results are presented as mean ± standard deviation and medians. The distribution of data was screened using histograms and Q-Q plots. To compare two groups, Mann-Whitney U test was used, and to compare more than two groups, the Kruskal-Wallis’s test was used followed by the Dunn-Bonferroni post hoc test for pairwise comparison. For categorical data, proportions are presented, and Fisher’s exact test was used to compare groups.

To better classify the patients according to organic and functional characteristics associated with frailty, we performed a cluster analysis. Initially, an analysis using the hierarchical method (square of the Euclidean distance) was performed to obtain the number of possible clusters, followed by a K-means clustering algorithm using the standardized values (z-score) of the following variables: age, preoperative hemoglobin, handgrip, MNA®, average gait speed, DASI, Dutch Exertion Fatigue Scale (DEFS), screening for depressive symptoms (PHQ-9), and handgrip strength.

All analyzes were performed using the IBM Corp. Released 2017, IBM SPSS Statistics for Windows, version 25.0, Armonk, NY: IBM Corp. software considering a significance level of *P* < 0.05.

## RESULTS

### Clinical Characteristics

A total of 117 patients were included in the study, 39 underwent CABG (33.3%) and 78 underwent valve surgery (66.7%). Table 1 shows the clinical characteristics of all patients according to the type of cardiac surgery. Table 2 shows the clinical characteristics of all patients according to Fried’s frailty phenotype^[^7^]^. The mean age of those considered frail according to Fried’s criteria was 60.43 ± 9.5 years, but 51% of those patients were under 60 years old.

**Table 1:** Clinical characteristics of the cohort.

		All patients	CABG	Valve surgery	*P*-value
					(CABG *vs.* valve surgery)
Female		45.3%	33.3%	51.3%	0.078
Age, years		58.82 ± 11.8	62.54 ± 8.5	56.96 ± 12.8	0.006
Weight, kg		75.16 ± 14.4	78.23 ± 11.3	73.63 ± 15.5	0.071
Stature, m		1.63 ± 0.1	1.63 ± 0.1	1.63 ± 0.1	0.855
Body mass index		28.26 ± 5.4	29.24 ± 4.6	27.77 ± 5.7	0.164
Ejection fraction		57.00 ± 11.9	54.62 ± 14.0	58.24 ± 10.6	0.125
Preoperative hemoglobin		12.72 ± 1.7	12.85 ± 1.7	12.66 ± 1.6	0.161
Preoperative creatinine		1.08 ± 0.4	1.05 ± 0.2	1.09 ± 0.4	0.557
Frailty	Non-frail	6.8%	12.8%	3.8%	0.105
	Pre-frail	49.6%	53.8%	47.4%	
	Frail	43.6%	33.3%	48.7%	
Systemic arterial hypertension		78.6%	94.9%	70.5%	0.002
Diabetes mellitus		29.9%	59.0%	15.4%	< 0.001
Smoke		13.7%	23.1%	9.0%	0.016
Previous cardiac operation		10.3%	0.0%	15.4%	0.008
Stroke		11.1%	7.7%	12.8%	0.540
Renal dysfunction		4.3%	5.1%	3.8%	0.747
Atrial fibrillation		20.5%	2.6%	29.5%	< 0.001
Peripheral arterial disease		3.4%	10.3%	0.0%	0.011

CABG=coronary artery bypass grafting

**Table 2:** Clinical characteristics of all patients according to Fried’s criteria of frailty.

	Non-frail	Pre-frail	Frail	*P*-value
Female	37.5%	34.5%	58.8%	0.034
Age, years	66.00 ± 6.1	56.41 ± 13.6	60.43 ± 9.5	0.042
Weight, kg	74.86 ± 12.3	75.49 ± 12.7	74.84 ± 16.6	0.971
Stature, m	1.63 ± 0.1	1.65 ± 0.1	1.60 ± 0.09	0.013
Body mass index	28.14 ± 3.9	27.30 ± 4.2	29.36 ± 6.5	0.138
Ejection fraction	58.75 ± 9.4	57.22 ± 11.3	56.44 ± 13.2	0.864
Preoperative hemoglobin	12.93 ± 1.1	12.98 ± 1.5	12.40 ± 1.9	0.192
Preoperative creatinine	1.05 ± 0.2	1.02 ± 0.2	1.14 ± 0.5	0.243
Unintentional weight loss	0.0%	6.9%	33.3%	0.001
Self-reported fatigue	0.0%	62.1%	88.2%	< 0.001
Low physical activity (IPAQ)	0.0%	69.0%	92.2%	< 0.001
Handgrip	34.38 ± 6.1	35.10 ± 10.1	24.02 ± 7.7	< 0.001
Gate speed (seconds)	4.97 ± 0.6	4.64 ± 0.9	6.70 ± 2.9	< 0.001
Duke Activity Status Index	37.29 ± 9.0	26.68 ± 11.9	16.37 ± 8.9	< 0.001
Dutch Exertion Fatigue Scale	10.25 ± 2.2	14.40 ± 5.9	21.39 ± 8.4	< 0.001
Mini Nutritional Assessment	12.50 ± 1.7	12.28 ± 2.0	11.33 ± 2.1	0.030
Patient Health Questionnaire-9 (or PHQ-9)	1.88 ± 1.7	3.67 ± 4.6	8.14 ± 6.2	< 0.001
Systemic arterial hypertension	62.5%	74.1%	86.3%	0.141
Diabetes mellitus	25.0%	31.0%	29.4%	0.936
Smoke habit	12.5%	22.4%	3.9%	0.032
Previous cardiac operation	0.0%	6.9%	15.7%	0.248
Stroke	0.0%	8.6%	15.7%	0.448
Renal dysfunction	0.0%	0.0%	9.8%	0.049
Atrial fibrillation	0.0%	15.5%	29.4%	0.074
Peripheral arterial disease	0.0%	3.4%	3.9%	0.851

IPAQ=International Physical Activity Questionnaire

The cardiopulmonary bypass time (minutes) for CABG and valve surgeries was 101.9 ± 42.3 minutes and 125.9 ± 58.0 minutes, respectively (*P* = 0.023). The aorta cross-clamping time was 77.4 ± 27.9 minutes and 96.9 ± 43.05 minutes for CABG and valve surgeries, respectively (*P* = 0.004). Overall, the postoperative in-hospital mortality was 14.5%. The postoperative in-hospital mortality rates for CABG and valve surgeries were 10.3% and 16.7%, respectively (*P* = 0.417).

The operative characteristics and postoperative hospital outcomes according to Fried’s frailty phenotype are shown in Table 3. The postoperative in-hospital mortality was 0% for non-frail, 15.5% for those deemed pre-frail, and 15.70% for the frail (*P* = 0.712).

**Table 3:** Operative characteristics and postoperative outcomes of all patients according to Fried’s frailty phenotype.

		Non-frail	Pre-frail	Frail	*P*-value
Cardiopulmonary bypass time (min)		100.63 ± 27.7	114.64 ± 54.7	124.43 ± 56.7	0.419
Aortic cross-clamping time (min)		83.88 ± 22.9	85.84 ± 37.6	96.65 ± 43.4	0.327
Operation	Valve surgery	37.5%	63.8%	74.5%	0.105
	CABG	62.5%	36.2%	25.5%	
Hospital death		0%	15.50%	15.70%	0.712
Time of invasive ventilation (h)		13.41 ± 8.7	32.60 ± 82.0	87.0 ± 223.6	0.039
Intensive care time (days)		3.39 ± 2.0	3.68 ± 4.4	7.32 ± 12.7	0.084
Invasive mechanical ventilation > 48 h		0%	10.30%	17.60%	0.375
Systemic inflammatory response		0%	5.20%	2.0%	0.716
Any postoperative infection		0.0%	6.9%	19.6%	0.109
Urinary infection		0%	0%	9.80%	0.049
Superficial wound infection		0%	0%	2.0%	0.504
Pneumonia		0%	3.40%	5.90%	0.765
Blood stream infection		0%	3.40%	3.90%	0.851
Mediastinitis		0%	0%	5.90%	0.182
Sepsis		0%	0%	7.80%	0.113
Acute kidney injury		0%	5.20%	15.70%	0.156
Stroke		0%	0%	2.0%	0.504
Circulatory shock		0%	12.10%	19.60%	0.310

CABG=coronary artery bypass grafting

**Fig. 1:**
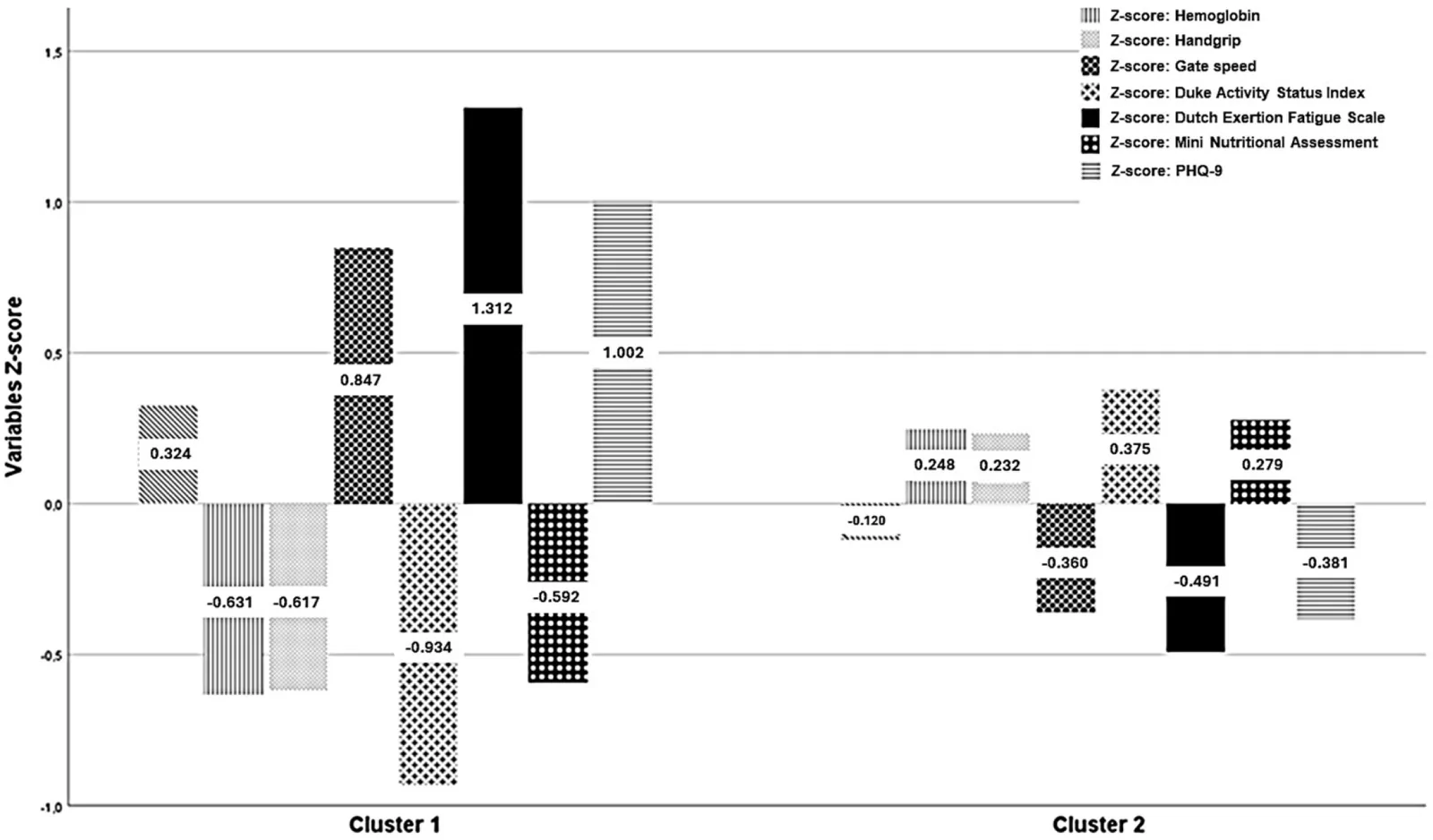
Cluster center of the variables included in the cluster analysis. PHQ-9=Patient Health Questionnaire-9.

The hierarchical cluster analysis resulted in two clusters (cluster 1 and cluster 2). The results of the K-means clustering algorithm with two clusters are shown in Figure 1 and Table 4. The variable with the smallest contribution (F-test) to the clustering algorithm was age, and the greatest contribution was provided by the variable DEFS. Clinical characteristics of both clusters are shown in Table 5.

**Table 4:** Contribution of each variable in the K-means cluster analysis (analysis of variance).

Z-score	Mean square	df	F-test	*P*-value
Age, years	4.665	1	4.845	0.030
Hemoglobin	18.326	1	22.499	< 0.001
Handgrip	17.095	1	20.373	< 0.001
Gate speed	34.499	1	53.301	< 0.001
Duke Activity Status Index	40.596	1	63.267	< 0.001
Dutch Exertion Fatigue Scale	77.057	1	222.467	< 0.001
Mini Nutritional Assessment	17.973	1	21.015	< 0.001
Patient Health Questionnaire-9 (PHQ-9)	45.317	1	69.992	< 0.001

**Table 5:** Clinical characteristics of the patients in the frailty-phenotype clusters.

		Cluster 1	Cluster 2	*P*-value
Female		63.6%	38.1%	0.014
Age, years		62.61 ± 9.43	57.33 ± 12.41	0.030
Weight, kg		74.27 ± 16.11	75.52 ± 13.73	0.674
Stature, m		1.58 ± 0.08	1.65 ± 0.09	0.001
Body mass index		29.22 ± 6.39	27.88 ± 4.93	0.228
Ejection fraction		56.27 ± 12.24	57.30 ± 11.89	0.680
Preoperative hemoglobin		11.65 ± 1.96	13.15 ± 1.34	< 0.001
Preoperative creatinine		1.23 ± 0.53	1.01 ± 0.31	0.007
Frailty (Fried)	Non-frail	0.0%	9.5%	< 0.001
	Pre-frail	15.2%	63.1%	
	Frail	84.8%	27.4%	
Unintentional weight loss		36.4%	10.7%	0.002
Self-reported fatigue		93.9%	59.5%	< 0.001
Low physical activity (IPAQ)		93.9%	66.7%	0.001
Handgrip		23.76 ± 8.75	32.76 ± 10.06	< 0.001
Gate speed (seconds)		7.58 ± 3.15	4.77 ± 1.02	< 0.001
Duke Activity Status Index		11.33 ± 5.08	27.46 ± 11.18	< 0.001
Dutch Exertion Fatigue Scale		27.36 ± 6.33	13.15 ± 3.78	< 0.001
Mini Nutritional Assessment		10.58 ± 2.18	12.39 ± 1.82	< 0.001
Patient Health Questionnaire-9 (PHQ-9)		11.09 ± 6.45	3.30 ± 3.53	< 0.001
Systemic arterial hypertension		97.0%	71.4%	0.001
Diabetes mellitus		27.3%	31.0%	0.284
Smoke habit		9.1%	15.5%	0.610
Previous cardiac operation		24.2%	4.8%	0.004
Stroke		18.2%	8.3%	0.188
Renal dysfunction		9.1%	2.4%	0.135
Atrial fibrillation		30.3%	16.7%	0.128
Peripheral arterial disease		3.0%	3.6%	0.885

IPAQ=International Physical Activity Questionnaire

The proportion of female patients in cluster 1 was significantly higher compared to cluster 2 (63.6% *vs.* 38.1%, *P* = 0.014). However, most of the variables associated with the frailty phenotype were significantly different between clusters 1 and 2 analyzing gender separately (Table 6).

**Table 6:** Variables used in cluster analysis, according to gender.

	Female			Male		
	Cluster 1	Cluster 2	*P*-value	Cluster 1	Cluster 2	*P*-value
Age, years	61.8 ± 9.6	55.7 ± 10.5	0.038	64.0 ± 9.3	58.3 ± 13.4	0.171
Preoperative hemoglobin	11.16 ± 1.7	12.79 ± 1.7	< 0.001	12.5 ± 2.1	13.4 ± 1.4	0.212
Preoperative creatinine	1.23 ± 0.6	0.93 ± 0.4	0.046	1.23 ± 0.2	1.07 ± 0.2	0.017
Unintentional weight loss	42.9%	0.0%	< 0.001	25.0%	17.3%	0.682
Self-reported fatigue	95.2%	62.5%	0.008	91.7%	57.7%	0.043
Low physical activity (IPAQ)	90.5%	59.4%	0.027	100.0%	71.2%	0.054
Handgrip	19.1 ± 5.8	25.6 ± 5.6	< 0.001	31.8 ± 7.0	37.1 ± 9.7	0.035
Gate speed (seconds)	8.3 ± 3.7	5.3 ± 1.6	0.001	6.35 ± 1.4	4.46 ± 0.9	0.001
Duke Activity Status Index	11.8 ± 5.4	25.0 ± 10.9	< 0.001	10.5 ± 4.6	29.0 ± 11.1	< 0.001
Dutch Exertion Fatigue Scale	27.2 ± 7.1	14.4 ± 4.0	< 0.001	27.7 ± 4.8	12.40 ± 3.5	< 0.001
Mini Nutritional Assessment	10.7 ± 2.1	13.0 ± 1.3	< 0.001	10.4 ± 2.4	12.02 ± 2.0	0.018
Patient Health Questionnaire-9 (PHQ)-9	11.2 ± 6.5	4.0 ± 3.8	< 0.001	10.8 ± 6.6	2.9 ± 3.3	0.002

IPAQ=International Physical Activity Questionnaire

Differently from the frailty phenotypes according to Fried et al.^[^7^]^, the cluster analysis provided cluster phenotypes more clearly associated with the postoperative outcomes, mainly postoperative in-hospital mortality (Table 7). In Fried’s phenotype, valve surgery is prevalent in the phenotypes of higher mortality (pre-frail and frail, Table 4), but the distribution of CABG and valve surgery was similar between cluster phenotypes 1 and 2. In cluster 1, 84.8% were frail and 15.2% were pre-frail, according to Fried et al.^[^7^]^.

**Table 7:** Operative characteristics and postoperative outcomes of each frailty-phenotype cluster.

		Cluster 1	Cluster 2	*P*-value
Cardiopulmonary bypass time (min)		135.15 ± 53.01	111.19 ± 53.64	0.031
Aortic cross-clamping time (min)		104.94 ± 40.80	84.71 ± 37.90	0.012
Invasive ventilation (h)		97.00 ± 242.09	38.50 ± 111.00	0.190
Intensive care time (days)		7.89 ± 14.21	4.21 ± 5.85	0.158
Operation	Valve surgery	75.8%	63.1%	0.276
	CABG	24.2%	36.9%	
Hospital death		27.3%	9.5%	0.020
Invasive mechanical ventilation > 48 h		18.2%	10.7%	0.357
Systemic inflammatory response		6.1%	2.4%	0.316
Any postoperative infection		24.2%	7.1%	0.022
Urinary infection		9.1%	2.4%	0.135
Superficial wound infection		3.0%	0.0%	0.282
Pneumonia		6.1%	3.6%	0.620
Blood stream infection		9,10%	1.2%	0.067
Mediastinitis		6.1%	1.2%	0.191
Sepsis		9.1%	1.2%	0.067
Acute kidney injury		21.2%	4.8%	0.011
Stroke		3.0%	0.0%	0.282
Circulatory shock		24.2%	10.7%	0.081

CABG=coronary artery bypass grafting

Considering all patients, in-hospital mortality in cluster 1 was 27.3% *vs.* 9.5% in cluster 2 (*P* = 0.020). However, postoperative in-hospital mortality for valve surgery in cluster 1 was 24% *vs.* 13.2% in cluster 2 (*P* = 0.329), and 37.5% in cluster 1 *vs.* 3.2% in cluster 2 for CABG (*P* = 0.022).

## DISCUSSION

The present study did not intend to identify isolated risk factors for postoperative hospital mortality but to verify if a more comprehensive evaluation of frailty, using variables to characterize dysfunction in domains usually relate to frailty, would better discriminate the frailty phenotype and its association with in-hospital mortality in adults who need one of the two most common cardiac operations. Therefore, our results showed that even though patients suffering from coronary artery disease are quite distinct from those with valve disease, regarding many anthropometric and clinical characteristics, they were similar regarding most of the variables associated with frailty according to Fried et al.^[^7^]^, and consequently, the distribution of frailty in both groups was similar. In addition, we observed that a considerable proportion of those considered frail were under 60 years old and that there was no association between frailty, according to Fried et al.^[^7^]^, and postoperative in-hospital mortality.

However, using a more comprehensive evaluation exploring dysfunction in domains affected by chronic heart conditions, we were able to identify two cluster phenotypes of frailty. Cluster 1 was associated with significantly higher rates of postoperative in-hospital mortality, acute renal dysfunction, and postoperative infections. In cluster 1, all patients were pre-frail or frail according to the phenotype model^[^7^]^, most were female with lower levels of hemoglobin and higher levels of creatinine, muscle weakness, worse cardiovascular performance and nutritional status, and lower physical activity. However, most of these aspects remained significantly different between clusters 1 and 2 comparing same gender patients (Table 6).

Frailty may be conceptualized as the loss of harmonic interaction between genetic, biological, functional, cognitive, psychological, and socioeconomic domains, in which multimorbidity and polypharmacy are the cause, as well as an effect. Nowadays there are two dominant models to assess frailty: the phenotype model and the cumulative deficit model. In the phenotype model^[^7^]^, the presence of three or more of the following criteria characterizes frailty: exhaustion, weight loss, weakness/loss of muscular strength, reduced gait speed, and reduced energy/physical activity. The cumulative deficit model considers an accumulation of deficits, including clinical signs and symptoms, diseases, and disability^[^1^]^. Thus, the recognition of frailty can be approximated by collecting information on performance, mobility, cognition, nutritional status, comorbidities, and general health status^[^9^]^.

The negative impact of frailty on the postoperative course after cardiovascular procedures has been demonstrated^[^10^]^. However, the best strategy for frailty assessment in candidates for surgical procedures still lacks consensus, especially if we consider that the tools for frailty assessment were designed for elderlies^[^11^]^.

Spiers et al.^[^12^]^, in their review of 268 publications - most using samples of mixed patients aged under and over 60 years -, did not identify proper tools designed to identify frailty exclusively in younger populations. The authors concluded that frailty measures have predictive validity in younger populations, but further evidence is needed to draw clear conclusions about the validity of these measures across the adult age spectrum.

In Brazil, the mean age of patients undergoing CABG is below 65 years^[^13^]^, and it is below 60 years for those undergoing valve surgery^[^14^]^, imposing more difficulties for the proper assessment of frailty in such patients.

Frailty and chronic illnesses are often treated as different entities, nevertheless, both are related and overlap^[^3^]^. In addition, we must consider not only the functional impairment caused by chronic illnesses but also the burden of psychological distress and low educational and socioeconomic status on frailty trajectories^[^15^]^. Thus, strategies to better identify frailty at any age in individuals with multiple morbidities and/or chronic diseases are necessary, mainly in the younger population.

The assessment of functional classification according to the New York Heart Association (NYHA) may be significantly influenced by the subjectivity of the physician and the patient. Similarly, the assessment of exhaustion and the physical activity level as proposed by Fried et al.^[^7^]^ may also suffer from considerable subjectivity. Our results with DEFS and DASI suggest that both are more appropriate tools for assessing levels of exhaustion and level of physical activity.

To date, we have not found studies in which DASI and DEFS were used as a tool for the assessment of domains related to frailty, especially in cardiac surgery. However, DASI is suggested by the Brazilian perioperative cardiac surgery guideline^[^16^]^ and by the American College of Cardiology^[^17^]^ as a way of estimating the preoperative functional capacity of patients. Wijeysundera et al.^[^18^]^ observed that a self-reported functional capacity (DASI) worse than a score of 34 was associated with increased odds of postoperative mortality or myocardial infarction. Additionally, Nepomuceno et al.^[^19^]^, studying outpatients with heart failure, found that an increase in the DEFS correlates with worsening of the NYHA class in patients with heart failure.

Sarcopenia is categorized into primary and secondary sarcopenias^[^20^]^, the former occurs with aging, and the latter is caused by systemic diseases; among these, there are the cardiovascular ones^[^21^]^, and they have a negative prognostic factor for patients undergoing cardiac surgery^[^22^]^. Even though depression and frailty are commonly associated^[^23^]^, there is evidence that perioperative depression by itself may increase postoperative mortality in patients undergoing cardiac surgery^[^24^]^. Similarly, malnutrition has been associated with mortality after cardiac surgery as an independent predictor^[^25^]^, and preoperative anemia, which may be a consequence of malnutrition and/or chronic disease, is also independently associated with adverse outcomes after cardiac surgery^[^26^]^. However, more data on the influence of preoperative nutritional rehabilitation on the outcomes of patients undergoing cardiovascular surgery are necessary.

### Limitations

The limitations of this study are associated with the small sample size and the clinical heterogeneity among patients. Furthermore, there is a lack of conclusive studies on the validity of the tools we used for the characterization of the domains associated with frailty (MNA®, gait speed, DASI, DEFS, PHQ-9, handgrip, and respiratory pressures) to obtain the clusters.

Even though such limitations, our study shows that frailty is not an uncommon condition in patients with heart disease in our environment, even in the younger, and we suggest that the criteria proposed by Fried et al.^[^7^]^ may not be suitable to evaluate frailty as a preoperative risk assessment in such a population. In addition, the present study may encourage future studies to investigate the best tools for characterizing frailty in patients undergoing cardiovascular operations, principally in non-elderly patients.

## CONCLUSION

In conclusion, we observed a considerable proportion of patients with Fried’s frailty phenotype in patients undergoing CABG or valve surgery, many of them under 60 years old, but those frailty phenotypes were not associated with postoperative hospital outcomes. However, the association of frailty phenotype with higher rates of postoperative in-hospital mortality was evident when additional criteria were used to assess the domains associated with frailty.

## Data Availability

The authors declare that the data will be available only upon request to the authors.

## Artificial Intelligence Usage

The authors declare that no artificial intelligence tools were used in the preparation, collection, or analysis of results, nor in the preparation of the manuscript.
